# *Marinobacter* sp. from marine sediments produce highly stable surface-active agents for combatting marine oil spills

**DOI:** 10.1186/s12934-017-0797-3

**Published:** 2017-11-02

**Authors:** Noura Raddadi, Lucia Giacomucci, Grazia Totaro, Fabio Fava

**Affiliations:** Department of Civil, Chemical, Environmental and Materials Engineering (DICAM), Alma Mater Studiorum-University of Bologna, Bologna, Italy

**Keywords:** Surface-active compounds, *Marinobacter* sp., Marine oil spills bioremediation, Biodispersants, Stability

## Abstract

**Background:**

The application of chemical dispersants as a response to marine oil spills is raising concerns related to their potential toxicity also towards microbes involved in oil biodegradation. Hence, oil spills occurring under marine environments necessitate the application of biodispersants that are highly active, stable and effective under marine environment context. Biosurfactants from marine bacteria could be good candidates for the development of biodispersant formulations effective in marine environment. This study aimed at establishing a collection of marine bacteria able to produce surface-active compounds and evaluating the activity and stability of the produced compounds under conditions mimicking those found under marine environment context.

**Results:**

A total of 43 different isolates were obtained from harbor sediments. Twenty-six of them produced mainly bioemulsifiers when glucose was used as carbon source and 16 were biosurfactant/bioemulsifiers producers after growth in the presence of soybean oil. Sequencing of 16S rRNA gene classified most isolates into the genus *Marinobacter*. The produced emulsions were shown to be stable up to 30 months monitoring period, in the presence of 300 g/l NaCl, at 4 °C and after high temperature treatment (120 °C for 20 min). The partially purified compounds obtained after growth on soybean oil-based media exhibited low toxicity towards *V. fischeri* and high capability to disperse crude oil on synthetic marine water.

**Conclusions:**

To the best of our knowledge, stability characterization of bioemulsifiers/biosurfactants from the non-pathogenic marine bacterium *Marinobacter* has not been previously reported. The produced compounds were shown to have potential for different applications including the environmental sector. Indeed, their high stability in the presence of high salt concentration and low temperature, conditions characterizing the marine environment, the capability to disperse crude oil and the low ecotoxicity makes them interesting for the development of biodispersants to be used in combatting marine oil spills.

**Electronic supplementary material:**

The online version of this article (10.1186/s12934-017-0797-3) contains supplementary material, which is available to authorized users.

## Introduction

Biosurfactants (BS) are amphipathic compounds produced by a variety of microorganisms. These compounds could be of low-molecular weight type, which are generally glycolipids and lipopeptides, and high-molecular weight type which are mainly lipopolysaccharides, lipoproteins or a combination of both. The high-molecular weight biosurfactants, frequently called bioemulsifiers (BE), are associated with production of stable emulsions, but do not always exhibit lowering of the surface or interfacial tension [1, 2]. The broad range of applications of these compounds has been documented by several patents on their use. Specifically, in 2006, 250 patents have been deposited worldwide and were mainly related to the use of BE/BS in petroleum (33%) or cosmetic (15%) industries; in medicine (12%) and in bioremediation (11%) [3]. Specifically, in bioremediation processes they play a crucial role in enhancing the bioavailability of hydrophobic compounds like hydrocarbons. Nonetheless, their application in response to oil spills in marine environment is not a common practice and chemical dispersants are currently the only products used [4]. The application of chemical dispersants is rising, however, some concerns due to their toxicity and negative effects on the activity of natural oil-degrading microorganisms [4]. Therefore, microbial surfactants, owing to their biodegradability and low toxicity [2, 5, 6], could be an alternative to their chemical counterparts for the development of biodispersants. The marine environment including oceans or oil reservoirs are characterized by high salinity reaching more than 150 g/l of NaCl [7] and quite low temperature that can range between 1 and 10 °C [8, 9]. Given the need for surface-active compounds with potential application for bioremediation under marine environmental conditions, interest in the selection of biosurfactants-producing marine microbes has been increasing. Microbes from marine habitats represent indeed an excellent opportunity to discover novel biosurfactants that are likely to function in marine ecosystems characterized by temperature extremes and low water activity [10, 11].

In this work, we aimed to (1) establish a collection of marine bacteria able to produce surface-active compounds after growth on soybean oil and/or glucose-based media and (2) characterize the produced compounds in terms of activity and stability also under challenging conditions.

## Methods

### Bacterial isolation

Marine sediment samples were collected from three Mediterranean harbors in the south of Italy in October 2013. Bacterial isolation was performed by spreading serial dilutions of grinded sediment samples in sterile saline solution (30 g/l NaCl in distilled water) on agar plates of modified mineral salt medium (mMSM) containing 1%_w/v_ of glucose as the major carbon source. The mMSM has the following composition (g/l): Na_2_HPO_4_, 0.7; KH_2_PO_4_, 0.9; NaNO_3_, 2; MgSO_4_·7H_2_O, 0.4; CaCl_2_·2H_2_O, 0.1; NaCl, 30 and 2 ml of trace element solution (per liter, 2 g FeSO_4_·7H_2_O, 1.5 g MnSO_4_·H_2_O, 0.6 g (NH_4_)_6_Mo_7_O_24_·4H_2_O). The pH of the medium was adjusted to 7.0 before autoclaving and solid medium was prepared by adding 15 g/l of agar.

### Screening for biosurfactant/bioemusifier production

Pure isolated strains, obtained by three successive streakings on mMSM agar plates, were inoculated into mMSM broth with 1%_w/v_ of glucose or 2%_v/v_ of soybean oil as carbon source and the flasks were incubated at 30 or 20 °C on a rotary shaker (150 rpm). After bacterial growth, the cell-free culture supernatants were recovered by centrifugation (10,000 rpm, 10 min, 4 °C), filter-sterilized (0.22 µm) and subjected to BS/BE production screening using three methods. Solutions of 0.5%_w/v_ of sodium dodecyl sulphate (SDS) and Tween 80 were used as positive controls; while deionized water and noninoculated growth medium as negative controls.

*Emulsification activity* (*EA*) was determined as described by [12] using 2 ml of culture supernatant and 2 ml of the tested organic solvent (*n*-hexane, toluene). The emulsification index value was recorded after 24 h (EI_24_) and expressed as the percentage of the height of the emulsion layer to the total height.

All screening assays were performed in triplicate as independent experiments.

*Drop collapse* The test was carried out as follows: 40 µl of the cell free supernatant was aliquoted as a droplet onto Parafilm^®^ (Parafilm M, Germany) surface; the flattening and the spreading of the droplet was followed over 10 min and recorded by visual inspection. If the drop remained beaded, the assay was scored negative, and if the drop collapsed, the result was positive.

*Interfacial surface tension (IFT)* of the cell-free culture supernatant was measured with a Drop Shape Analyzer—DSA30 (KRUSS GmbH, Germany) working in the pendant drop mode. The surface tension was calculated from at least three measurements by the instrument software using the Young–Laplace method. Deionized water and sterile medium were used as references for high IFT liquids, while SDS as low IFT reference.

### Stability of bioemulsifiers

Stability studies were performed using cell-free culture supernatants. In order to assess the effect of salinity on the bioemulsifier activity, culture supernatants were supplemented with NaCl up to a final concentration of 300 g/l and the emulsifying activity was measured as described above. To evaluate the stability of the bioemulsifier at high temperatures, culture supernatants were subjected to autoclaving (121 °C for 20 min); the samples were then cooled to room temperature and the emulsification indexes (EI_24_) were measured and compared to the corresponding values before treatment. The activity and stability of the bioemulsifiers was evaluated at 4 °C using culture supernatants and solvents that were cooled to 4 °C and incubated at 4 °C after vortexing. Furthermore, the stability of the emulsions produced was monitored for up to 30 months after incubation at room temperature or 4 °C. All the experiments were carried out in triplicate. The results were reported as residual emulsification activity (REA) (%) expressed as follows:

$$REA \left( \% \right) = \frac{{EI_{t} }}{{EI_{24} }} \times 100$$

where EI_t_ is the EI (%) value at time t; and compared with the positive controls.

### Partial purification of biosurfactants/bioemulsifiers

After growth in mMSM supplemented with 2%_w/v_ soybean oil, culture supernatants were recovered by centrifugation (10,000 rpm, 10 min, 4 °C), filter-sterilized (0.22 µm filter), acidified with 6 N hydrochloric acid solution to pH 2.0 and allowed to settle at 4 °C overnight. The precipitated crude BE/BS collected by centrifugation (10,000 rpm, 20 min, 4 °C) were dried (4–5 h at 50 °C) and either used for FT-IR analysis or dissolved in distilled H_2_O and adjusted to pH 7.0 using 1 N NaOH for CMCs determination. The dissolved BE/BS were also subjected to dialysis (cut-off 14,000 Da, Sigma-Aldrich Co.) before using for ecotoxicological analysis or oil displacement test.

### Preliminary chemical characterization of biosurfactants/bioemulsifiers

The infra-red spectra (FT-IR) of the dried BE/BS were recorded over the wavenumber range 450–4000 cm^−1^ using a Perking Elmer Spectrum One FT-IR spectrometer (transmission mode) after grinding with KBr to form a very fine powder (1/100 ratio) and compressing into a thin pellet. Thirty-two scans were taken for each spectrum at a resolution of 2 cm^−1^.

### CMC measurements

Partially purified biosurfactants obtained by acid precipitation were dissolved in distilled H_2_O at 4 g/l and then the solutions were diluted to concentration ranging from 0.10 to 3.7 g/l. The surface tension of each dilution was measured as described above. IFT values were plotted as a function of the logarithm of biosurfactant concentration and CMC was then calculated as the intersection point between the baseline of minimal surface tension and the slope where surface tension shows a linear decline.

### Evaluation of acute toxicity to the luminescent bacterium *Vibriofischeri*

Acute toxicity assays of partially purified BE/BS were performed according to the standard protocol for the Microtox basic test [5] using 1 mg/ml for bioemulsifiers, or the specific CMC for biosurfactants. Results were reported as EC_50_, the effective concentration of partially purified biosurfactant that caused a 50% reduction in the amount of luminescence emitted by the bacterial suspension after 30 min of exposure.

### Dispersant assay

The dispersant capacity of the BS/BE was evaluated by performing the oil displacement test carried out as follows: 20 μl of light crude oil were slowly dropped onto the surface of 40 ml of artificial seawater in a Petri dish (9 cm in diameter) until covering the entire surface area of the water. Afterwards, 20 μl of partially purified BS/BE solution prepared at the CMC concentration or at 1 g/l were added onto the surface of the oil layer. The mean diameter of the clear zones was recorded from triplicate experiments. A solution of 1%_w/v_ SDS or distilled water were used as positive and negative controls, respectively.

### PCR amplification and sequencing of 16S rRNA gene

PCR amplification of 16S rRNA gene was performed on DNA extracted from each isolate using the GenElute Bacterial Genomic Kit (Sigma) and bacterial universal primers 27f (5′-AGAGTTTGATCCTGGCTCAG-3′) and 1492r (5′-CTACGGCTACCTTGTTACGA-3′) with the following reaction conditions: 1 × PCR buffer (Invitrogen, Milan, Italy), 1.5 mM MgCl_2_, 0.2 mM of each dNTP, 0.4 μM of each primer, 1 U of Taq polymerase in a final volume of 50 μl. Initial denaturation at 95 °C for 4 min was followed by 30 cycles consisting of denaturation at 95 °C for 1 min, annealing at 55 °C for 1 min, and extension at 72 °C for 2 min. A final extension at 72 °C for 10 min was added. The amplification products were sequenced by the company Macrogen (South Korea). Sequences were checked for chimeras using DECIPHER software [13], identified using the BLASTn and a Neighbor-Joining phylogenetic tree was then built using MEGA6 [14], computing the evolutionary distances using the Jukes–Cantor method. Sequences were deposited in the GenBank database under accession numbers MF382054 to MF382079.

## Results and discussion

### Bacterial isolation and selection of biosurfactant/bioemulsifier-producers

After bacterial growth in mMSM broth with glucose, 43 pure isolates selected based on their different colony morphology after several successive streakings on mMSM agar medium, were screened for the BS/BE production. The results of drop collapse, IFT and EI_24_ of different organic solvents for the producer isolates are reported in Table 1. Among the 43 isolates screened, 26 showed an EI_24_ higher than 50% in at least one of the solvents tested. The maximum emulsifying activity was observed between 48 and 72 h of incubation. In particular, the highest EI_24_ was observed towards toluene and equal to 75.0 ± 1.7%. Cell-free culture supernatants of most isolates exhibited a weak drop collapse activity and only few of them were able to slightly decrease the medium surface tension (from 74.7 ± 0.2 to 60.2 ± 1.6 mN/m, Table 1). It has been reported that low-molecular weight BS are able to reduce the surface tension of aqueous media to around 40 mN/m, while the high molecular weight BE often form and stabilize emulsions without remarkable surface tension reduction [15, 16]. These results suggest that under these experimental conditions, mainly BE were produced.

**Table 1:** List of the 26 bacterial surface-active compounds producers selected among 43 isolates obtained from marine sediments and grown in mMSM with 1%_w/v_ glucose as carbon source

Isolate ID	16SrDNA Accession No	Closest type strain (GenBank Accession No)	16S rDNA identity (%)	Drop collapse	IFT (mN/m)	EI_24_ (%) Hexane	EI_24_ (%) Toluene
M11.30	MF382057	*Bacillus hwajinpoensis SW*-*72 (NR_025264)*	99	−	71.6 ± 0.2	16.0 ± 1.7	49.8 ± 7.6
M21.30	MF382056	*Bacillus hwajinpoensis SW*-*72 (NR_025264)*	98	−	68.4 ± 0.1	25.0 ± 2.4	62.7 ± 0.9
G3.20	MF382058	*Halomonas alkaliantarctica strain CRSS (NR_114902.1)*	99	+	71.9 ± 1.7	45.7 ± 6.1	67.0 ± 1.7
G5.20	MF382059	*Halomonas venusta strain DSM 4743 (NR_042069.1)*	98	+	72.0 ± 0.1	65.0 ± 1.7	70.0 ± 0.0
G6.20	MF382060	*Halomonas alkaliantarctica strain CRSS (NR_114902.1)*	99	+	66.5 ± 1.6	67.0 ± 0.0	67.0 ± 0.0
P11.20	MF382061	*Halomonas titanicae BH1 (NR_117300)*	99	−	71.5 ± 0.2	66.7 ± 0.0	69.0 ± 0.0
G2.30	MF382062	*Thalassospira xiamenensis DSM 17429 (CP004388)*	99	+	72.6 ± 1.2	33.3 ± 4.7	70.0 ± 0.0
G1.30	MF382065	*Marinobacter hydrocarbonoclasticus ATCC 49840T (NR_074619)*	99	−	74.4 ± 0.4	63.3 ± 4.7	68.3 ± 2.4
G19.30	MF382076	*Marinobacter similis strain A3d10 (KJ547704)*	99	−	74.6 ± 0.1	62.0 ± 0.0	75.0 ± 1.7
M15.20	MF382063	*Marinobacter salarius strain R9SW1(KJ547705)*	99	−	66.7 ± 0.5	58.6 ± 4.9	62.1 ± 4.9
M18.20	MF382073	*Marinobacter salarius strain R9SW1(KJ547705)*	99	−	70.9 ± 0.8	63.9 ± 0.8	66.7 ± 0.0
M20.20	MF382064	*Marinobacter salarius strain R9SW1(KJ547705)*	99	−	72.1 ± 0.1	56.9 ± 0.3	62.7 ± 0.9
M22.20	MF382072	*Marinobacter salarius strain R9SW1(KJ547705)*	99	+	66.5 ± 1.7	62.0 ± 1.7	62.0 ± 0.0
M24.20	MF382074	*Marinobacter salarius strain R9SW1(KJ547705)*	99	−	70.3 ± 1.7	45.0 ± 7.1	63.0 ± 6.7
M27.20	MF382071	*Marinobacter salarius strain R9SW1(KJ547705)*	98	+	69.6 ± 0.6	57.0 ± 0.0	55.9 ± 5.8
M28.20	MF382075	*Marinobacter salarius strain R9SW1(KJ547705)*	99	−	71.9 ± 0.1	61.0 ± 1.5	63.7 ± 2.4
M1.30	MF382066	*Marinobacter flavimaris strain SW*-*145 (NR_025799.1)*	99	−	71.9 ± 0.1	59.2 ± 5.8	33.3 ± 0.0
M10.30	MF382067	*Marinobacter guineae strain M3B (NR_042618.1)*	99	+	72.0 ± 0.1	60.0 ± 0.0	67.0 ± 0.0
M13.30	MF382069	*Marinobacter sediminum strain R65 (NR_029028.1)*	99	−	71.9 ± 0.1	61.0 ± 1.5	66.1 ± 0.8
M17.30	MF382077	*Marinobacter sediminum strain R65 (NR_029028.1)*	99	+	72.3 ± 0.0	63.3 ± 0.0	70.0 ± 0.0
M24.30	MF382078	*Marinobacter sediminum strain R65 (NR_029028.1)*	99	+	69.7 ± 1.1	61.7 ± 2.4	66.7 ± 0.0
M25.30	MF382070	*Marinobacter sediminum strain R65 (NR_029028.1)*	99	+	60.2 ± 1.6	63.0 ± 0.0	73.0 ± 0.0
M26.30	MF382068	*Marinobacter guineae strain M3B (NR_042618.1)*	99	+	72.0 ± 0.1	53.0 ± 3.3	68.0 ± 1.7
M27.30	MF382079	*Marinobacter salarius strain R9SW1(KJ547705)*	96	−	69.2 ± 1.2	57.6 ± 1.4	50.0 ± 4.7
M16.30	MF382055	*Marinobacter salarius strain R9SW1(KJ547705)*	99	+	67.1 ± 1.0	51.7 ± 5.0	66.7 ± 0.0
P7.30	MF382054	*Marinobacter adhaerens strain HP15 (NR_074765)*	100	−	66.8 ± 1.6	64.4 ± 1.54	68.3 ± 0.0

### 16S rRNA gene sequencing of the bioemulsifier-producing strains

The molecular identification of the 26 BS/BE-producing isolates was performed by sequencing the 16S rRNA gene and comparing the sequences to the NCBI 16S rRNA database. The isolates were shown to belong to the genera *Bacillus*, *Thalassospira*, *Halomonas* and *Marinobacter* (Table 1, Additional file 1: Figure S1). The results of almost full 16S rRNA gene sequence (1445 bp-long) comparison indicated that isolates M21.30 and M11.30 have the highest sequence similarities of 98 and 99% with *Bacillus hwajinpoensis* strain SW-72 isolated from sea water [17]. Phylogenetic analyses of the 1362 bp-long 16S rRNA gene sequence placed isolate G2.30 within the genus *Thalassospira*. A sequence similarity of 99% was found with *Thalassospira xiamensis* strain M-5 isolated from the surface water of a waste oil pool at the oil storage dock in the city of Xiamen [18] and *Thalassospira xianhensis* strain P-4 isolated from oil-polluted saline soil [19], respectively. At a sequence similarity of 99% of the 1416 bp-long 16S rRNA gene sequence, the closest type strain relative of isolate P11.20 was *Halomonas titanicae* BH1 isolated from the Titanic ship wreck in Atlantic Ocean [20]. The 16S rRNA gene sequences of isolates G3.20 and G6.20 showed similarity of 99% with strains *Halomonas alkaliantarctica* strain CRSS obtained from saline lake in Antarctica [21] while that of isolate G5.20 showed similarity of 98% with the validated species *Halomonas venusta* strain DSM 4743 [22]. With regard to the isolates belonging to the genus *Marinobacter*, with the exception of isolate P7.30 that exhibited 100% similarity with *Marinobacter adhaerens* strain HP15 [23], each of the other sequences showed a similarity of 99% with type strain of different species of the genus isolated from marine environment [24, 25]. Many bacteria within these genera could not be identified to the species level based only on 16S rRNA gene sequences [26–28], hence further analyses need to be performed in order to establish the exact phylogenetic positions of the isolates, which is beyond the aim of this study. The obtained results are in line with previous surveys where these bacterial genera have been reported to be indigenous to marine environments [17, 27–30]. The production of surface-active molecules from marine bacteria has been reported by several authors [31–34]. However, to the best of our knowledge, there have been no reports on the characterization of surface-active molecules production from the *Marinobacter* and *Thalassospira* although bacteria within these genera have been often retrieved from hydrocarbon-enriched marine communities [35–37]. It is also of note that, we were able to obtain bacteria mainly from the *Marinobacter* genus starting from sediments subjected to hydrocarbons contamination in the harbors, supporting previous studies reporting on the specialization of bacteria from this genus in lipids and hydrocarbons metabolism [29].

### Effect of salinity and temperature on emulsification activity

**Fig. 1:**
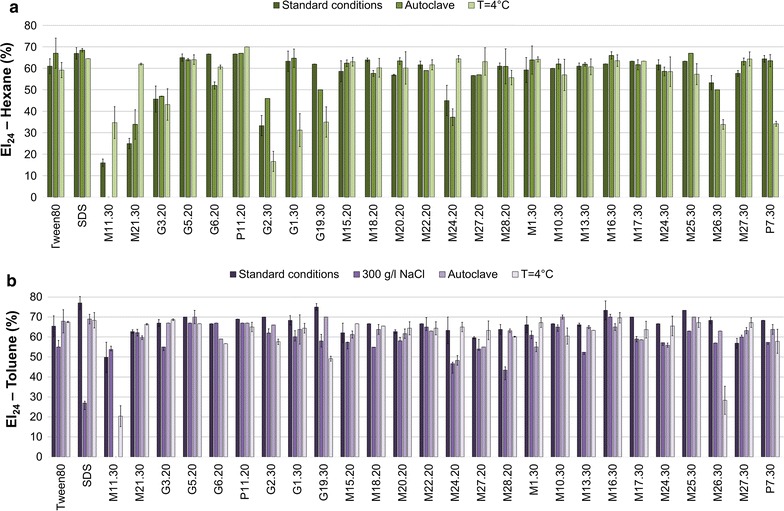
Emulsification index of hexane (**a**) and toluene (**b**) solvents under standard conditions or extremes of temperature and water activity of the crude bioemulsifiers produced after bacterial growth in mMSM with 1%_w/v_ glucose as major carbon source. Each bar represents the mean of three replicates of EI_24_ (%). Error bars illustrate experimental errors (± SD) calculated from three independent experiments

The activity of the crude BEs was evaluated from cell-free culture supernatants, recovered after growth on glucose-based media, after exposure to low water activity as well as to high or low temperature (Fig. 1). The emulsification activity was not evaluated towards hexane when high salt concentration, i.e. 300 g/l of NaCl, was tested, since even positive controls did not exhibit emulsifying activity towards the solvent under such conditions. Using toluene as organic solvent, in the presence of 300 g/l of NaCl, the common chemical surfactant SDS exhibited a reduced emulsifying activity as shown by the decrease of the EI_24_ to 27 ± 3.3% from 77 ± 3.3% under standard conditions. On the opposite, with the exception of supernatants from isolates G19.30, M24.20, M28.20 and M13.30, no significant impact on the emulsifying potential was observed; and most of the isolates exhibited an EI_24_% equal to that recorded under standard conditions (Fig. 1a). The effect of thermal treatment on the emulsifier activity showed that, with few exceptions, no appreciable changes has occurred in the emulsification capacity of most supernatants using both aromatic and aliphatic hydrocarbon solvents (Fig. 1). In specific, a complete loss of emulsification capacity of both solvents was observed in the case of isolate M11.30 after autoclaving (121 °C, 20 min); while a decrease of hexane emulsification in the case of supernatants from isolates G1.30, G2.30, G19.30, M26.30 and P7.30 and of that of toluene by G19.30, M11.30 and M26.30 was recorded at 4 °C. On the other hand, the emulsification activity of isolates M24.20, M11.30 and M21.30 towards hexane was shown to be higher at 4 °C compared to the standard conditions. Higher emulsification activity compared to the standard conditions was also recorded for autoclaved supernatant of G2.30 towards hexane. Gutiérrez et al. [33] reported that heat (100 °C) and acid conditions (0.1 N) increase the relative activity of the bioemulsifiers produced by *Halomonas* sp. They referred to this mechanism as heat activation of polymeric emulsifiers, explained by the fact that this treatment may have induced the release of a higher number of emulsifying moieties from these biopolymers that would contribute to their increased emulsifying capacity. BE that are not affected by NaCl concentrations up to 300 g/l have been described from *Paenibacillus* sp. #510 [38]. Dubey et al. [39] evaluated the effect of increasing temperature and salt concentrations on biosurfactant activities of *Pseudomonas aeruginosa* strain-PP2 and *Kocuria turfanesis* strain-J and found that both bioemulsifiers retained their activity up to 200 g/l NaCl and to 121 °C. Purified trehalolipid biosurfactant produced by a marine *Rhodococcus* sp. produced emulsions that were stable to a wide range of temperatures (20–100 °C) and NaCl concentrations (50–250 g/l) [40]. Currently, we are aware of one study reporting on stable biosurfactant activity at low temperature in the case of *Brevibacterium luteolum*. However, in that case the assay was performed under slightly different conditions, i.e. evaluation of the surface activity was performed at room temperature after incubating the biosurfactants at 4 °C for 24 h [41], while in our case the emulsions were let to form at 4 °C and using cold culture supernatant and solvents, resulting in stabilizing the formed emulsion. The fact that the produced bioemulsifiers were active at low temperature and high salt concentration is an optimal trait to be considered when application under seawater conditions are targeted.

The obtained results showed that there is no clear correlation between the bacterial growth temperature and the optimal temperature of the bioemulsifier activity. Indeed, independently of the fact that bacteria were grown at 30 or 20 °C, the emulsification capability was strain-specific. The fact that also bioemulsifiers obtained from isolates grown at 30 °C were highly active at 4 °C is highly interesting from an industrial viewpoint. Indeed, growing bacteria at 20 °C would result in extra energy consumption related to bioreactor cooling.

### Emulsions stability as a function of time

The stability of the emulsions produced under standard screening conditions or after exposure to water or heat stress was evaluated as a function of incubation time (Table 2, Additional file 2: Table S1). The emulsions formed showed a high stability at room temperature, maintaining up to 100% of the original emulsification activity of both solvents over a period of 18 months at 4 °C and even in the presence of 300 g/l of NaCl. Moreover under standard conditions, it was possible to monitor and record a REA of 100% up to 30 months of incubation in the case of BE produced by *Marinobacter* sp. strains compared to a REA of 65 or 77.5% recorded for the controls Tween 80 and SDS, respectively (Table 2). Extended stability in time has been previously observed for the BE produced by marine *Pedobacter* sp. strain MCC-Z where the emulsions remained stable for 4 months [42] and for the bioemulsifiers produced by a marine *Antarctobacter* [32]. However, to the best of our knowledge, no reports on extended stability under low water activity, after thermal treatment conditions or 30 months are available and especially from nonpathogenic marine bacteria such as *Marinobacter* sp.

**Table 2:** Residual emulsification activity (REA) (%) after 18 or 30 months* of incubation of bioemulsifiers produced by the isolates grown on mMSM with 1%_w/v_ glucose as carbon source and positive controls

Bacterial isolate/positive control	Hexane			Toluene			
	Standard conditions	Autoclave	4 °C	Standard conditions	300 g/l NaCl	Autoclave	4 °C
Tween 80	*65.00 ± 0.00	*0.00 ± 0.00	98.44 ± 0.55	*86.36 ± 0.00	*0.00 ± 0.00	*0.00 ± 0.00	79.93 ± 10.82
SDS	*77.50 ± 0.00	*0.00 ± 0.00	95.58 ± 3.65	*65.73 ± 0.00	*14.30 ± 0.00	*0.00 ± 0.00	83.05 ± 4.66
*Bacillus* sp. M11.30	0.00 ± 0.00	NA	0.00 ± 0.00	43.27 ± 2.16	23.21 ± 4.42	NA	0.00 ± 0.00
*Bacillus* sp. M21.30	*64.09 ± 4.06	93.64 ± 6.14	97.84 ± 0.77	*18.59 ± 0.93	87.50 ± 4.13	83.72 ± 5.55	96.86 ± 0.61
*Halomonas* sp. G3.20	57.28 ± 2.86	86.44 ± 4.32	39.06 ± 1.95	82.92 ± 4.15	51.14 ± 2.56	0.00 ± 0.00	85.37 ± 6.55
*Halomonas* sp. G5.20	86.84 ± 0.36	58.59 ± 2.93	96.25 ± 0.36	67.97 ± 4.89	55.97 ± 2.80	23.04 ± 1.15	87.93 ± 0.00
*Halomonas* sp. G6.20	55.56 ± 2.78	0.00 ± 0.00	79.61 ± 1.20	85.00 ± 4.25	79.29 ± 3.96	99.36 ± 4.97	91.28 ± 0.00
*Halomonas* sp. P11.20	95.89 ± 1.25	89.55 ± 4.48	84.73 ± 1.39	56.42 ± 2.82	93.28 ± 4.66	38.52 ± 1.93	94.48 ± 1.95
*Thalassospira* sp. G2.30	0.00 ± 0.00	0.00 ± 0.00	84.82 ± 6.31	0.00 ± 0.00	100.00 ± 0.00	0.00 ± 0.00	85.25 ± 8.68
*Marinobacter* sp. G1.30	*92.12 ± 7.89	82.37 ± 1.82	89.09 ± 6.29	*80.36 ± 7.58	89.00 ± 7.24	81.70 ± 5.68	93.69 ± 0.30
*Marinobacter* sp. G19.30	0.00 ± 0.00	0.00 ± 0.00	46.13 ± 2.10	0.00 ± 0.00	0.00 ± 0.00	0.00 ± 0.00	87.12 ± 8.12
*Marinobacter* sp. M15.20	*74.71 ± 9.25	53.38 ± 1.32	76.39 ± 1.54	*90.02 ± 6.68	88.91 ± 7.25	88.51 ± 1.09	95.69 ± 3.66
*Marinobacter* sp. M18.20	*78.25 ± 4.84	97.42 ± 0.08	80.10 ± 1.63	*90.00 ± 0.00	71.98 ± 9.96	88.05 ± 1.18	94.74 ± 0.00
*Marinobacter* sp. M20.20	*25.21 ± 1.26	74.48 ± 8.04	78.98 ± 0.05	*97.93 ± 2.92	93.44 ± 2.24	89.53 ± 3.42	92.58 ± 0.74
*Marinobacter* sp. M22.20	87.11 ± 4.36	56.50 ± 2.82	95.13 ± 3.64	100.00 ± 0.00	86.54 ± 4.33	84.33 ± 4.22	100.00 ± 0.00
*Marinobacter* sp. M24.20	*85.38 ± 5.52	0.00 ± 0.00	98.06 ± 0.08	*90.55 ± 9.63	0.00 ± 0.00	0.00 ± 0.00	95.55 ± 3.46
*Marinobacter* sp. M27.20	*100.00 ± 0.00	33.96 ± 1.70	90.27 ± 7.03	*89.88 ± 9.26	11.57 ± 0.58	72.73 ± 3.64	93.33 ± 4.39
*Marinobacter* sp. M28.20	*64.48 ± 9.82	35.93 ± 9.80	81.85 ± 6.03	*95.24 ± 3.64	35.63 ± 0.11	77.81 ± 0.80	87.23 ± 2.35
*Marinobacter* sp. M1.30	*98.94 ± 0.00	88.95 ± 7.85	55.60 ± 6.85	*88.96 ± 2.47	91.98 ± 0.12	90.78 ± 0.71	92.37 ± 3.35
*Marinobacter* sp. M10.30	91.49 ± 7.47	95.77 ± 4.79	79.60 ± 5.28	96.77 ± 0.00	93.24 ± 4.66	89.29 ± 4.46	77.07 ± 2.65
*Marinobacter* sp. M13.30	*69.84 ± 9.26	79.41 ± 3.07	89.34 ± 4.56	*96.51 ± 2.50	92.27 ± 2.62	91.84 ± 1.17	87.22 ± 4.25
*Marinobacter* sp. M16.30	85.93 ± 7.59	83.20 ± 4.16	94.44 ± 4.04	91.10 ± 5.86	85.25 ± 4.26	93.38 ± 4.67	93.27 ± 2.19
*Marinobacter* sp. M17.30	*90.23 ± 4.51	87.84 ± 2.31	100.00 ± 0.00	*85.71 ± 4.29	100.00 ± 0.00	92.75 ± 1.93	96.20 ± 2.94
*Marinobacter* sp. M24.30	*11.19 ± 0.43	87.04 ± 5.24	64.11 ± 9.44	*95.00 ± 0.00	97.54 ± 0.07	82.97 ± 6.41	95.52 ± 3.25
*Marinobacter* sp. M25.30	69.14 ± 6.43	84.11 ± 4.21	62.20 ± 3.54	83.58 ± 4.18	84.33 ± 4.22	90.17 ± 4.51	90.79 ± 1.12
*Marinobacter* sp. M26.30	0.00 ± 0.00	50.00 ± 2.50	49.32 ± 3.32	63.59 ± 7.82	82.59 ± 4.13	71.68 ± 3.58	41.38 ± 2.07
*Marinobacter* sp. M27.30	*93.40 ± 3.01	87.04 ± 5.24	93.91 ± 5.53	*87.05 ± 8.21	97.22 ± 3.93	93.83 ± 2.33	92.94 ± 0.39
*Marinobacter* sp. P7.30	*100.00 ± 0.00	75.58 ± 4.76	97.62 ± 3.37	*99.26 ± 1.04	93.15 ± 2.65	94.59 ± 0.21	78.33 ± 7.07

*NA* not applicable (the stability was not monitored when no EI_24_ was recorded)

### Effect of carbon source on biosurfactant/bioemulsifiers production and stability

**Fig. 2:**
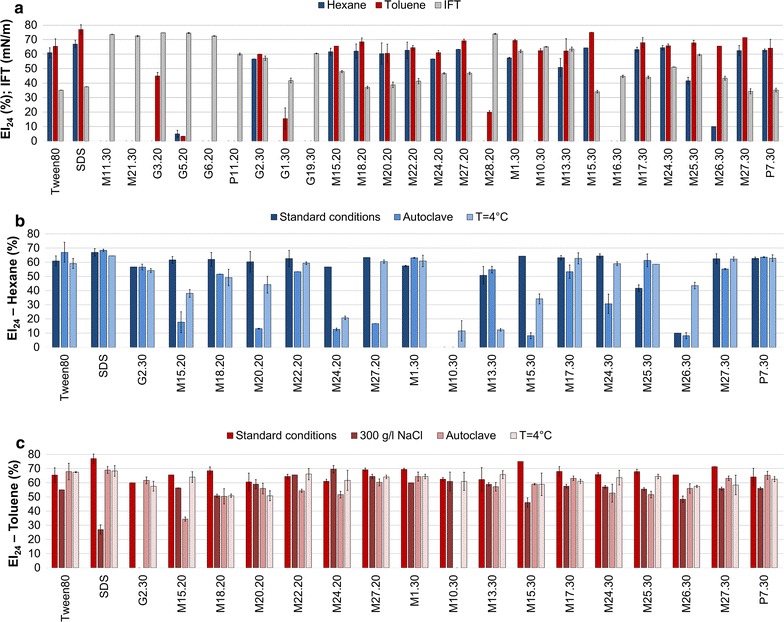
Evaluation of the surface activities of the BS/BE after bacterial growth in mMSM with 2%_v/v_ soybean oil as major carbon source. IFT (mN/m) and emulsification index under standard conditions (**a**); emulsification index of hexane (**b**) and toluene (**c**) solvents under extremes of temperature and water activity. Each bar represents the mean of three measurements of IFT or three replicates of EI_24_ (%)

The 26 selected isolates were also tested for their growth and surface-active molecules production on a relatively cheap and highly available carbon source. All the isolates were able to grow on mMSM with 2%_v/v_ soybean oil as main carbon source and most of them produced surface-active molecules. In specific, 16 isolates including *Thalassospira* sp. G2.30 and 15 *Marinobacter* sp. exhibited significant emulsification activity (EI_24_ > 50%) towards at least one of the solvents tested and the highest emulsification index was equal to 71.4 ± 0.0% towards toluene (Fig. 2a). Cell-free culture supernatants of most isolates exhibited a high drop collapse activity (data not shown) and were able to decrease the medium surface tension from 74.66 ± 0.21 up to 34.35 ± 1.73 mN/m (Fig. 2a). These results suggest that most of the isolates were able to produce BE that are able to reduce the surface tension. The supernatants of isolates P11.20 and G19.30 did not exhibit bioemulsification activity towards the solvents tested. They however reduced only slightly the medium surface tension which suggests the production of BE that are not able to emulsify the toluene and hexane, since slight IFT reduction is often characteristic of BEs. The cell-free culture supernatant of isolate M16.30 did not emulsify the solvents tested but was able to exhibit an IFT value of 44.7 ± 0.8 mN/m (Fig. 2a).

In order to evaluate the stability of the produced emulsions, the cell free culture supernatants of BE producers were subjected to temperature (high and low) and water (300 g/l NaCl) stresses (Fig. 2b, c). Cell free-culture supernatants were able to highly emulsify toluene with the exception of those of isolates M10.30, M15.20 and G2.30 for which a significant decrease or loss of the activity was observed after autoclaving or in the presence of 300 g/l NaCl, respectively. The low temperature did not affect the emulsifying ability of the crude bioemulsifiers of almost all the isolates towards both solvents, except for M26.30 and M10.30, which exhibited a higher emulsification index of hexane at this temperature.

Furthermore, similarly to the BE produced after growth in glucose-based media, the produced emulsions were stable under the different conditions tested up to 18 months of incubation at room temperature retaining up to 100% of the activity displayed under standard conditions (Table 3).

**Table 3:** Residual activity (REA, %) after 18 months of incubation of crude bioemulsifiers produced by the isolates grown on mMSM with 2%_v/v_ soybean oil as main carbon source

Bacterial isolate	HEXANE			TOLUENE			
	Standard conditions	Autoclave	4 °C	Standard conditions	300 g/l NaCl	Autoclave	4 °C
*Thalassospira* sp. G2.30	58.82 ± 2.94	32.16 ± 1.99	85.69 ± 2.18	66.67 ± 3.33	0.00 ± 0.00	75.32 ± 7.27	92.48 ± 7.38
*Marinobacter* sp. M15.20	63.89 ± 8.48	0.00 ± 0.00	52.86 ± 9.28	97.37 ± 3.72	94.46 ± 4.29	86.81 ± 8.90	93.95 ± 5.58
*Marinobacter* sp. M18.20	76.35 ± 4.04	86.45 ± 4.83	76.97 ± 0.87	100.00 ± 0.00	66.77 ± 3.10	67.67 ± 8.79	98.33 ± 2.36
*Marinobacter* sp. M20.20	62.50 ± 3.13	0.00 ± 0.00	92.64 ± 4.63	82.59 ± 8.88	94.43 ± 0.44	58.57 ± 2.41	34.16 ± 6.55
*Marinobacter* sp. M22.20	67.52 ± 9.06	66.96 ± 3.35	90.16 ± 5.59	100.00 ± 0.00	77.47 ± 9.93	96.94 ± 0.22	94.87 ± 0.19
*Marinobacter* sp. M24.20	92.80 ± 6.45	86.21 ± 8.28	68.68 ± 3.89	100.00 ± 0.00	90.00 ± 4.50	93.41 ± 4.09	97.50 ± 3.54
*Marinobacter* sp. M27.20	88.30 ± 9.10	69.64 ± 7.58	63.80 ± 7.83	94.83 ± 0.58	78.95 ± 0.00	85.51 ± 4.78	96.75 ± 1.78
*Marinobacter* sp. M1.30	93.75 ± 8.84	86.02 ± 9.24	83.73 ± 7.57	87.83 ± 1.18	83.33 ± 4.06	90.00 ± 2.92	93.74 ± 6.03
*Marinobacter* sp. M10.30	NA	NA	92.86 ± 6.64	94.83 ± 0.58	65.61 ± 0.60	NA	69.03 ± 2.60
*Marinobacter* sp. M13.30	100.00 ± 0.00	52.00 ± 6.99	87.44 ± 6.22	87.30 ± 6.42	94.99 ± 0.05	82.79 ± 7.05	94.33 ± 1.19
*Marinobacter* sp. M17.30	87.62 ± 5.04	81.62 ± 9.36	89.67 ± 1.10	97.37 ± 3.72	83.77 ± 2.01	90.38 ± 5.75	90.38 ± 5.75
*Marinobacter* sp. M24.30	94.27 ± 2.26	40.89 ± 0.89	97.51 ± 3.52	0.00 ± 0.00	86.10 ± 0.55	88.56 ± 6.08	92.44 ± 7.44
*Marinobacter* sp. M25.30	0.00 ± 0.00	35.06 ± 2.61	85.29 ± 4.16	94.14 ± 5.85	35.94 ± 7.84	32.41 ± 6.55	92.11 ± 3.72
*Marinobacter* sp. M26.30	65.59 ± 1.52	0.00 ± 0.00	99.34 ± 0.94	92.65 ± 5.69	43.89 ± 6.20	67.29 ± 3.70	92.74 ± 6.87
*Marinobacter* sp. M27.30	95.37 ± 5.57	27.08 ± 7.75	97.37 ± 3.72	100.00 ± 0.00	87.87 ± 0.52	90.48 ± 2.24	87.76 ± 6.79
*Marinobacter* sp. P7.30	96.84 ± 1.38	90.99 ± 9.92	99.08 ± 1.30	93.99 ± 8.42	89.28 ± 1.48	95.16 ± 4.03	98.59 ± 0.98

### Preliminary chemical characterization

**Fig. 3:**
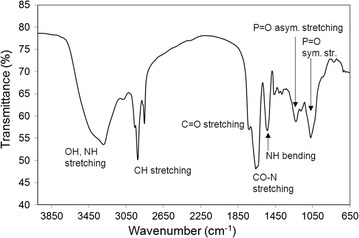
Example of a phospholipopeptide FTIR spectrum of partially purified BS/BE produced by one of the isolates (*Marinobacter* sp. M22.20) after growth in mMSM with 2%_v/v_ soybean oil as major carbon source; asym. and sym. str: asymmetric and symmetric stretching of phosphate anion

Partially purified BS/BE obtained by acid precipitation from cell-free culture supernatants of the isolates exhibiting significant surface activity after growth in soybean-based medium, were subjected to FT-IR analysis for the identification of the main functional groups present. All the samples show the main absorption bands of a polypeptide, that is to say amide I and II: at 1654 cm^−1^ and around 1540 cm^−1^ the stretching mode of the CO–N bond and the bending mode of NH bond are present. In some samples the band of CO–N has two peaks, therefore two conformation are probably present [43, 44]. In addition, all the spectra disclosed a broad stretching peak into the region 3100–3400 cm^−1^ characteristic of N–H and O–H groups. Absorptions around 2960 cm^−1^ are assigned to the symmetric C–H stretch of CH_2_ and CH_3_ groups of aliphatic chains. The asymmetric and symmetric stretching of phosphate anion can be found at almost 1236 and 1070 cm^−1^, respectively [45]. The typical carbonyl stretching group (C=O), indicating of an ester, is present in almost all samples at about 1740 cm^−1^. Therefore, the FT-IR spectra suggested that, with the exception of M16.30, M15.20 and M26.30 which seem to produce phosphopeptides BS, the produced surface active compounds belong to the phospholipopeptide class of BS for almost all the isolates tested (Fig. 3). Lipoprotein bioemulsifiers have been reported from *Pseudomonas nitroreducens* isolated from a mangrove ecosystem [46]. Protein surface-active compounds from *Acinetobacter* have been reported to highly emulsify organic solvents [47] while no reports are available on their ability to reduce surface tension. Anionic residues such as phosphates and sulfates have been reported to play an important role in the emulsification of *n*-hexadecane by the EPS produced by *Halomonas* sp. [48]. Hence, the absence of emulsification activity of the phosphopeptide-producer *Marinobacter* sp. M16.30 could be that the produced compound is of small molecular weight and therefore is not able to emulsify the organic solvents tested in this study.

### Critical micelle concentration

The determination of biosurfactants CMC is crucial since there is no further modification in the surfactant activity above this concentration. The CMCs were determined on partially purified biosurfactants obtained from cell-free culture supernatants (after growth in the presence of 2%_v/v_ soybean oil as major carbon source) through acid precipitation. The CMCs of the biosurfactants produced by the 11 isolates (that were able to reduce surface tension to values ≤ 45 mN/m) were estimated to range between 2520 and 620 mg/l among the different isolates (Table 4). These values are higher compared to those reported for chemical biosurfactants like Tween 80 (110 mg/l) [39] or Triton X-100 (150 mg/l) and to other marine bacteria such as *Streptomyces* sp. (36 mg/l) [49], *Brevibacterium luteolum* (40 mg/l) [41] and *Halomonas* sp. MB-30 (250 mg/l) [50]. On the other hand, they are in agreement with BE/BS produced by different bacterial species like the purified bioemulsifier produced by *Pedobacter* sp. strain MCC-Z, a glycolipids-protein complex, that showed high emulsifying activity (EI_24_ = 64%) and reduced the surface tension of water up to 41 mN/m with a critical micelle concentration value of 2600 mg/l [42]. The comparison with *Marinobacter* sp. is not possible since, to the best of our knowledge, no reports on characterization of BS produced by bacteria from this genus are available.

**Table 4:** Toxicity data obtained for the different BS/BE produced by the isolates after growth on mMSM with 2%_v/v_ soybean oil as carbon source

Sample	Concentration tested (mg/l)	EC_50_ (mg/l) after 30 min
SDS	1000	110.99 ± 16.36
*Marinobacter* sp. G1.30	620	3.10 ± 1.85
*Marinobacter* sp. M15.20	1000	380.70 ± 39.46
*Marinobacter* sp. M18.20	1620	194.05 ± 59.59
*Marinobacter* sp. M20.20	2350	ND
*Marinobacter* sp. M22.20	1560	ND
*Marinobacter* sp. M24.20	1320	24.13 ± 11.62
*Marinobacter* sp. M27.20	2070	113.54 ± 23.69
*Marinobacter* sp. M1.30	1000	58.94 ± 35.22
*Marinobacter* sp. M13.30	1000	31.91 ± 12.02
*Marinobacter* sp. M16.30	2520	ND
*Marinobacter* sp. M17.30	970	549.58 ± 59.10
*Marinobacter* sp. M24.30	1000	77.21 ± 6.96
*Marinobacter* sp. M25.30	1000	60.95 ± 20.57
*Marinobacter* sp. M26.30	2350	131.54 ± 14.90
*Marinobacter* sp. M27.30	1390	118.08 ± 19.67
*Marinobacter* sp. P7.30	1580	93.72 ± 35.22

The partially purified BS were used at their CMCs while a concentration of 1000 mg/l was tested in the case of BE and SDS. The values are those obtained from three independent experiments (± SD). ND: not determined, the increase in bioluminescence did not allow calculating the EC_50_ value

### Ecotoxicity assays

The toxicity of the BE/BS produced by the bacterial isolates and of the synthetic surfactant SDS was assessed using *V. fischeri* as an indicator microorganism. The partially purified BS were used at their CMCs while a concentration of 1000 mg/l was tested in the case of BE. The EC_50_ values obtained are shown in Table 4. After 30 min of exposure, the EC_50_ values of several (6/17) of the tested isolates were higher compared to that of SDS. Specifically, the EC_50_ of SDS used at 1000 mg/l was equal to 110.99 ± 16.36 mg/l while those of most of the isolates ranged between 113.54 ± 23.69 and 549.58 ± 59.10 mg/l. Interestingly, for three *Marinobacter* sp. isolates (M20.20, M22.20 and M16.30), it was not possible to calculate the EC_50_ value since an increase of the bioluminescence was observed; this indicates the nontoxic character of the tested biosurfactants. On the contrary, several isolates exhibited higher toxicity towards *V. fischeri* at the concentrations tested (Table 4). Although it is difficult to compare and discuss in deep the obtained data with those available in literature due to the lack of a unique way to report the results of ecotox assays towards *V. fischeri*, it is possible to conclude that the BE/BS from *Marinobacter* sp. isolates exhibit similar or less toxicity when compared with other microbial surface-active compounds. In similar assays, Lima et al. [5] reported EC_20_ values for different bacterial biosurfactants between 261 and 736 mg/l. Gudiña et al. [38] reported that *V. fischeri* bioluminescence was reduced by 29% after 30 min exposure to the bioemulsifier produced by *Paenibacillus* sp. at a concentration of 1000 mg/l. Franzetti et al. [51] reported a low toxicity for the bioemulsifier produced by *Variovorax paradoxus* 7bCT5 against *V. fischeri*, with 34 ± 2% bioluminescence inhibition after 15 min of exposure to the highest concentration tested (500 mg/l).

### Evaluation of the dispersant ability of the surface active compounds

**Fig. 4:**
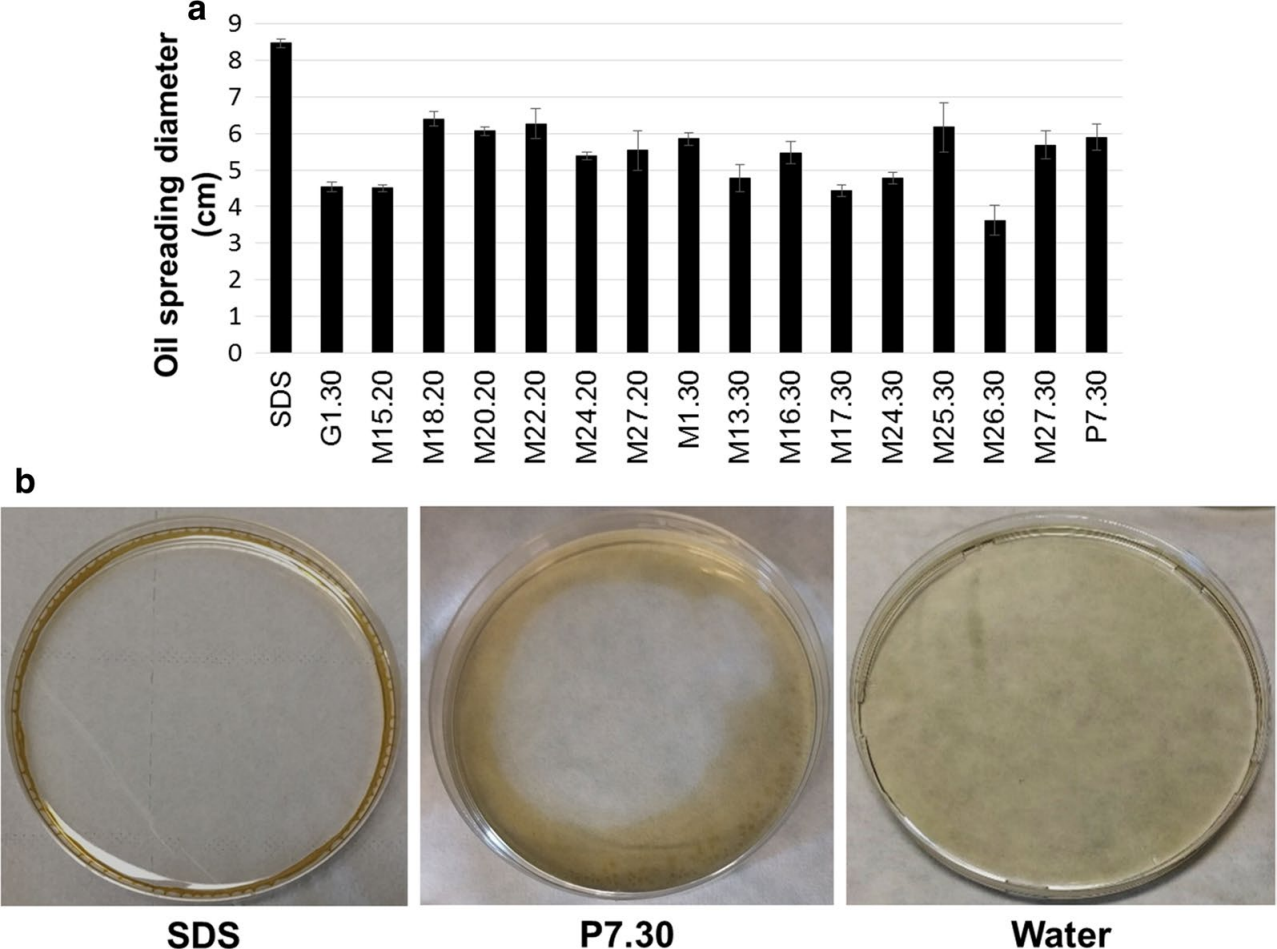
Crude oil dispersant activities in artificial seawater of partially purified biosurfactants/bioemulsifiers obtained from *Marinobacter* sp. isolates after growth in mMSM with 2%_v/v_ soybean oil as major carbon source. **a** Diameter of oil spreading; error bars illustrate experimental errors (± SD) calculated from three independent experiments; **b** Example of dispersant activities to crude oil with SDS, water or partially purified BS/BE isolated from *Marinobacter* sp. P7.30

Solutions of the partially purified surface-active compounds prepared at the same concentrations as those applied for ecotox assays were used in oil spreading test. Figure 4 illustrates the crude oil dispersant capacity of the BS/BE in artificial sea water. Compared to SDS used as control which exhibited a spreading diameter of 8.5 ± 0.1 cm, the solutions of the partially purified BS/BE showed spreading diameters ranging between 3.6 ± 0.4 and 6.4 ± 0.2 cm. The oil spreading ability observed here is higher compared to that reported by Sriram et al. [52] for lipopeptide BS produced by *Escherichia fergusonii*, which exhibited an oil spreading diameter of 2.66 ± 0.20 cm in the presence of 30 g/l NaCl. A higher spreading diameter i.e. 19 cm was recorded for a lipopeptide produced by *Bacillus atrophaeus* 5-2a but the assay was performed using paraffine oil [2].

## Conclusions

A collection of marine bacteria, mainly belonging to *Marinobacter* sp., able to produce BS/BE was established. The bioemulsifiers were active and stable under extremes of temperature, low water activity and up to 30 months of incubation. Further, they exhibited good environmental compatibility as shown by their low ecotoxicity and were able to disperse crude oil in artificial marine water. Owing to these characteristics, the produced BS/BE would be very interesting for developing biodispersant formulations to be applied for the bioremediation of marine oil spills. Furthermore, *Marinobacter* sp. are considered nonpathogenic; and hence are suitable for BS/BE large scale production. Studies towards evaluating the ecosustainability of pilot scale production are in progress.

## Additional files

**Additional file 1.** Phylogenetic affiliation of the almost entire 16S rRNA gene of the bacterial isolates constructed using MEGA6 package. Neighbor-Joining phylogenetic tree was built using MEGA6, computing the evolutionary distances using the Jukes–Cantor method.

**Additional file 2: Table S1.** Residual emulsification activity (REA) (%) under standard conditions after 18 of incubation of bioemulsifiers produced by the 14 isolates grown on mMSM with 1%_w/v_ glucose as carbon source.
