# Supplementary figures and images for: Marinobacter sp. from marine sediments produce highly stable surface-active agents for combatting marine oil spills
Source: Microb Cell Fact. 2017 Nov 2;16:186. doi: 10.1186/s12934-017-0797-3 (PMC5668961; doi:10.1186/s12934-017-0797-3)

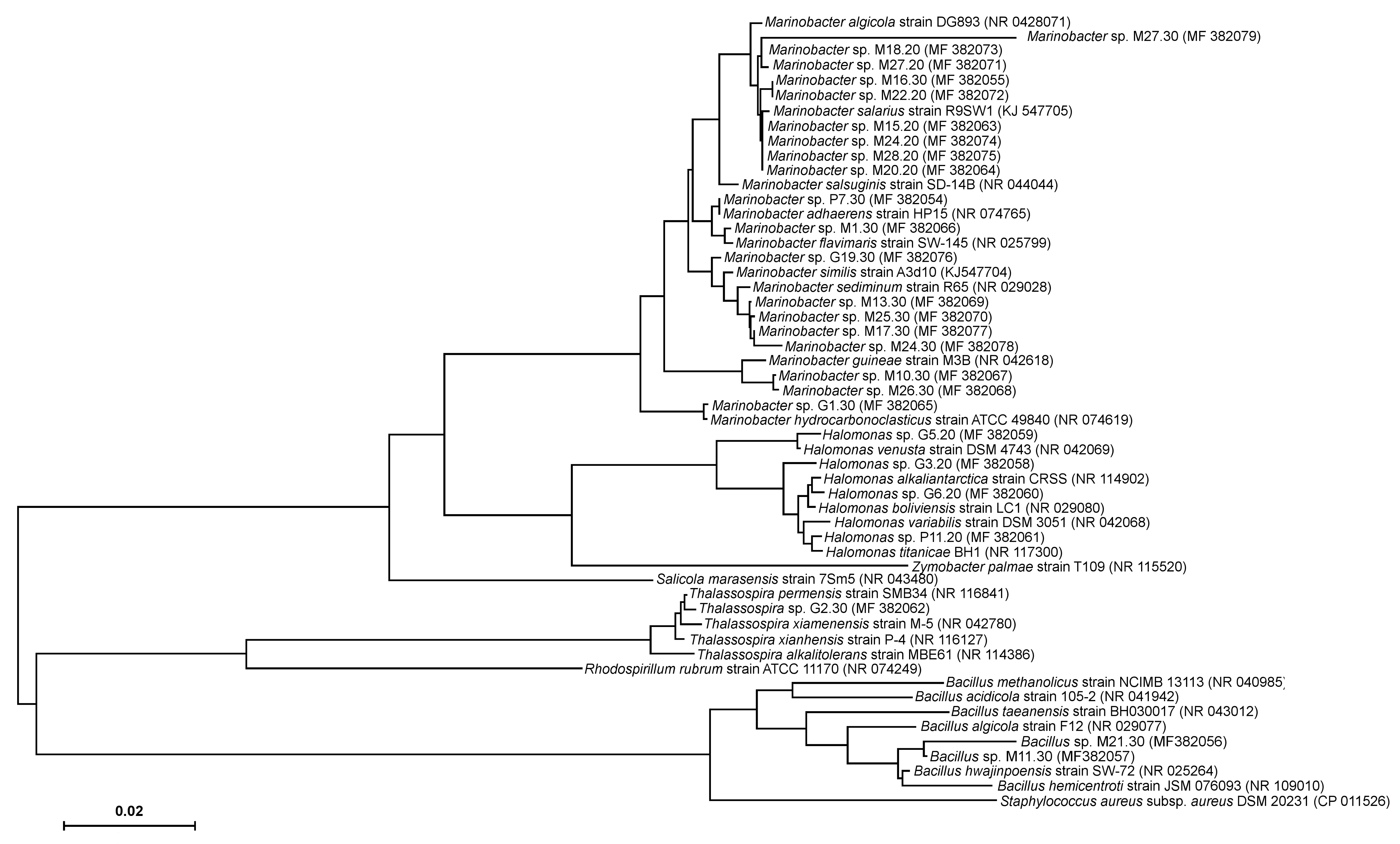

Supplement: Supplementary file 1 — Additional file 1. Phylogenetic affiliation of the almost entire 16S rRNA gene of the bacterial isolates constructed using MEGA6 package. Neighbor-Joining phylogenetic tree was built using MEGA6, computing the evolutionary distances using the Jukes–Cantor method. [file 12934_2017_797_MOESM1_ESM.tif]
