# Supplementary material for: Marinobacter sp. from marine sediments produce highly stable surface-active agents for combatting marine oil spills
Source: Microb Cell Fact. 2017 Nov 2;16:186. doi: 10.1186/s12934-017-0797-3 (PMC5668961; doi:10.1186/s12934-017-0797-3)
Supplement: Supplementary file 2 — Additional file 2: Table S1. Residual emulsification activity (REA) (%) under standard conditions after 18 of incubation of bioemulsifiers produced by the 14 isolates grown on mMSM with 1%w/v glucose as carbon source. [file 12934_2017_797_MOESM2_ESM.docx]

**Additional file 2: Table S1.** Residual emulsification activity (REA) (%) under standard conditions after 18 months of incubation of bioemulsifiers produced by the 14 isolates grown on mMSM with 1%_w/v_ glucose as carbon source.

| **Bacterial isolate** | **Hexane** | **Toluene** |
| --- | --- | --- |
| *Bacillus* sp. M21.30 | 70.85 ± 2.44 | 54.08 ± 0.53 |
| *Marinobacter sp.* G1.30 | 93.44 ± 4.13 | 95.05 ± 3.62 |
| *Marinobacter sp.* M15.20 | 81.94 ± 9.82 | 90.98 ± 8.04 |
| *Marinobacter sp.* M18.20 | 100.00 ± 0.00 | 92.50 ± 5.34 |
| *Marinobacter sp.* M20.20 | 79.82 ± 5.92 | 97.09 ± 0.10 |
| *Marinobacter sp.* M24.20 | 88.34 ± 2.36 | 87.89 ± 3.66 |
| *Marinobacter sp.* M27.20 | 100.00 ± 0.00 | 89.21 ± 10.69 |
| *Marinobacter sp.* M28.20 | 72.22 ± 7.86 | 94.12 ± 3.60 |
| *Marinobacter sp.* M1.30 | 97.05 ± 1.99 | 92.81 ± 4.64 |
| *Marinobacter sp.* M13.30 | 68.59 ± 2.72 | 100.00 ± 0.00 |
| *Marinobacter sp.* M17.30 | 95.86 ± 3.83 | 95.24 ± 4.76 |
| *Marinobacter sp.* M24.30 | 54.24 ± 9.72 | 96.77 ± 0.00 |
| *Marinobacter sp.* M27.30 | 95.47 ± 4.33 | 91.96 ± 6.13 |
| *Marinobacter sp.* P7.30 | 99.66 ± 0.48 | 99.54 ± 0.65 |
